# Changes in Metabolic Profiles during Acute Kidney Injury and Recovery following Ischemia/Reperfusion

**DOI:** 10.1371/journal.pone.0106647

**Published:** 2014-09-05

**Authors:** Qingqing Wei, Xiao Xiao, Paul Fogle, Zheng Dong

**Affiliations:** 1 Department of Cellular Biology and Anatomy, Medical College of Georgia, Georgia Regents University and Charlie Norwood VA Medical Center, Augusta, Georgia, United States of America; 2 Wuhan University, Wuhan, Hubei, China; 3 Metabolon Inc, Durham, North Carolina, United States of America; 4 Department of Nephrology, The Second Xiangya Hospital, Central South University, Changsha, Hunan, China; Center for Molecular Biotechnology, Italy

## Abstract

Changes of metabolism have been implicated in renal ischemia/reperfusion injury (IRI). However, a global analysis of the metabolic changes in renal IRI is lacking and the association of the changes with ischemic kidney injury and subsequent recovery are unclear. In this study, mice were subjected to 25 minutes of bilateral renal IRI followed by 2 hours to 7 days of reperfusion. Kidney injury and subsequent recovery was verified by serum creatinine and blood urea nitrogen measurements. The metabolome of plasma, kidney cortex, and medulla were profiled by the newly developed global metabolomics analysis. Renal IRI induced overall changes of the metabolome in plasma and kidney tissues. The changes started in renal cortex, followed by medulla and plasma. In addition, we identified specific metabolites that may contribute to early renal injury response, perturbed energy metabolism, impaired purine metabolism, impacted osmotic regulation and the induction of inflammation. Some metabolites, such as 3-indoxyl sulfate, were induced at the earliest time point of renal IRI, suggesting the potential of being used as diagnostic biomarkers. There was a notable switch of energy source from glucose to lipids, implicating the importance of appropriate nutrition supply during treatment. In addition, we detected the depressed polyols for osmotic regulation which may contribute to the loss of kidney function. Several pathways involved in inflammation regulation were also induced. Finally, there was a late induction of prostaglandins, suggesting their possible involvement in kidney recovery. In conclusion, this study demonstrates significant changes of metabolome kidney tissues and plasma in renal IRI. The changes in specific metabolites are associated with and may contribute to early injury, shift of energy source, inflammation, and late phase kidney recovery.

## Introduction

Acute kidney injury (AKI) is a major kidney disease characterized by a rapid decline of renal function. Clinically, renal ischemia/reperfusion is one of the main causes of AKI [1]. As the main function of kidney is to excrete the metabolic wastes and toxins, the loss of kidney function in AKI is expected to induce significant changes in various metabolites in the body, especially in the plasma of the blood [2]. Moreover, the change of specific metabolites may indicate the pathways that are critically important in the pathogenesis of renal IRI and subsequent recovery. In addition, the change of certain metabolites in the early phase of renal IRI may suggest their possibility of serving as biomarkers for the diagnosis of AKI.

Metabolomics is the measurement of metabolome in biological matrices, which provides a systematic view of the change of all metabolites in the physiological or pathological conditions. Recently after the establishment of metabolome databases in different systems, the global profiling of metabolites has been used in various disease models, including renal diseases, and is particularly useful to identify novel disease markers for diagnosis and new targets for drug discovery [2], [3]. Metabolomics has been introduced to study the changes in metabolism in AKI within the last a few years, especially in nephrotoxic models including those induced by gentamycin, cisplatin, nicotinic acid receptor agonist, melamine and cyanuric acid [2], [4]–[13]. In renal IRI, a recent study by Liu et. al. examined the changes of metabolites in a rat model using high-performance liquid chromatography coupled with mass spectrometry-based approach [14]. While the study by Liu et al. revealed interesting changes, how the changes are related to kidney injury was not fully analyzed. It is also unclear if there are significant metabolic changes during the kidney recovery or repair phase following renal IRI. In addition, the differential changes in the metabolme of plasma, kidney cortex, and medulla are unknown.

In this study, we analyzed the changes of metabolic profiles in kidney cortex, medulla, and serum after renal IRI in mice. The analysis covered the phases of initial kidney injury, peak injury, and the recovery. In addition to the overall change of metabolome, we also identified specific metabolites that showed early responses to ischemic injury and the perturbed metabolism pathways that are related to energy metabolism, osmotic regulation and the induction of inflammation.

## Materials and Methods

### Animals and renal ischemic surgery

C57BL/6 mice were originally from The Jackson Laboratory (Bar Harbor, ME) and bred in the animal facility of Charlie Norwood VA Medical Center. The mice were housed under 12 hr/12 hr dark and light cycle with free access to water and food. Male littermates of C57BL/6 mouse at around 9 weeks old were used for renal ischemia/reperfusion surgery and sham operation as described previously [15]–[17]. Briefly, the mice were anesthetized with 60 mg/kg of pentobarbital. The surgery was conducted on a homoeothermic pad with the animal body temperature controlled at 36.5°C. Flank incisions were made to expose the kidney and both of the renal pedicles were clamped with micro-aneurysm clips to block the blood flow. After 25 minutes of ischemia, the micro-aneurysm clips were released for the reperfusion. The mice under sham operation experienced the similar procedure except the renal ischemia. All the procedures performed for animal breeding, housing, surgeries were following the protocol approved by the Institutional Animal Care and Use Committee of the facility in Charlie Norwood VA Medical Center.

### Kidney sample collection

The mice were sacrificed at the designated reperfusion time. The kidneys were excised immediately and dissected on an ice plate to separate the cortical and medullar part. All the samples were freshly frozen in liquid nitrogen and stored in −80°C till analysis.

### Plasma sample collection

The whole blood was collected when sacrificing the mice and kept on ice. An equal volume of 9 mM EDTA in phosphate buffered saline was then added. After mixing, the samples were centrifuged at 1000 g for 15 minutes in 4°C and the supernatants were collected as plasma. All the samples were frozen in liquid nitrogen immediately and stored in −80°C till analysis.

### Renal function measurement

The whole blood was collected when sacrificing the mice and clotted in the room temperature. The samples were then centrifuged at 12000 g for 5 min in the room temperature and the supernatants were collected as serum. All the samples were kept in 4°C till use. The serum creatinine level was measured with Creatinine LiquiColor Test (Kinetic) from Stanbio Laboratory (Boerne, TX) following the instruction manual. The blood urea nitrogen (BUN) level was examined with Urea Nitrogen (BUN) Diacetylmonoxime Test Kit from Stanbio Laboratory (Boerne, TX) following the manual. Renal function was calculated according to the standard curve and compared with student t-test using Microsoft Excel 2010 and P<0.05 was considered as statistically significant difference.

### Global Metabolic Profiling

The global metabolic profiling was conducted by Metabolon, Inc. (Durham, NC). At the time of analysis samples were extracted and prepared for analysis using Metabolon's standard solvent extraction method. The extracted samples were split into equal parts for analysis on the gas chromatography/mass spectrometry (GC/MS) and liquid chromatography/mass spectrometry (LC/MS) platforms. Technical replicate samples created from a homogeneous pool containing a small amount of all study samples (“Client Matrix”) were also included. The instrument variability was determined by calculating the median relative standard deviation for the internal standards that were added to each sample prior to injection into the mass spectrometers. The overall process variability was determined by calculating the median relative standard deviation for all endogenous metabolites present in 100% of the Client Matrix samples, which are technical replicates of pooled client samples.

The informatics system used for sample management, data extraction and analysis consisted of four major components, the Laboratory Information Management System (LIMS), the data extraction and peak-identification software, the data processing tools for quality control and compound identification, and a collection of information interpretation and visualization tools for use by data analysts. The hardware and software foundations for these informatics components were the LAN backbone, and a database server running Oracle 10.2.0.1 Enterprise Edition.

The data extraction of the raw mass spec data files yielded information that could loaded into a relational database and manipulated without resorting to BLOB manipulation. Once in the database the information was examined and the appropriate quality control limits were imposed. The peaks were identified using Metabolon's proprietary peak integration software, and the component parts were stored in a separate and specifically designed complex data structure.

The compounds were identified by comparison to library entries of purified standards or recurrent unknown entities. The identification of known chemical entities was based on the comparison to metabolomic library entries of purified standards. As to this study, more than 1000 commercially available purified standard compounds had been acquired registered into LIMS for distribution to both the LC/MS and GC/MS platforms for determination of their analytical characteristics. The combination of chromatographic properties and mass spectra gave an indication of a match to the specific compound or an isobaric entity. Metabolon data analysts used proprietary visualization and interpretation software to confirm the consistency of peak identification among the various samples. The library matches for each compound were checked for each sample and corrected if necessary.

Two types of statistical analysis are performed: the significance test and the classification analysis. Welch's two sample t-test was used to show the pair-wise difference. For classification random forest analyses was used to create a set of classification trees based on continual sampling of the experimental units and compounds. The statistical analyses are performed with the program “R” http://cran.r-project.org/.

### Ethics

All the procedures performed for animal breeding, housing, surgeries were following the protocol approved by the Institutional Animal Care and Use Committee of the facility in Charlie Norwood VA Medical Center (Protocol No. 11-07-036).

## Results and Discussions

### Twenty-five minutes of bilateral renal ischemia induces recoverable acute kidney injury

**Figure 1:**
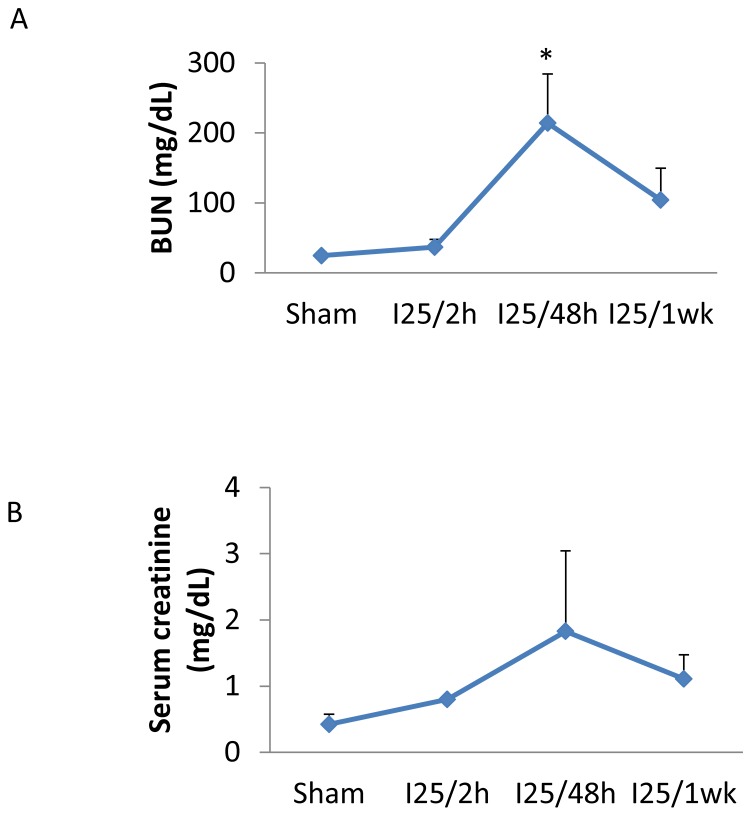
Renal function of C57BL/6 mice during recoverable renal IRI. Littermate C57BL/6 mice were subjected to sham operation or 25 minutes of bilateral renal ischemia with 2 hours, 48 hours, and 1 week of reperfusion. The serum samples were collected at sacrifice to monitor renal function by measuring: (A) blood urea nitrogen (BUN); (B) Serum creatinine. Data are means ±S.D. from 3 separate experiments. *, statistically significant difference comparing to the sham condition.

C57BL/6 mice were subjected to 25 minutes of bilateral renal ischemia followed by 2 hours, 48 hours and 1 week of reperfusion. The renal function was monitored by the BUN and the serum creatinine level (Fig. 1). Comparing to the sham operated mice (BUN 24.6±5.2 mg/dL, creatinine 0.42±0.16 mg/dL), there was slightly loss of renal function at 2 hours of reperfusion after ischemia, showing only marginal increases in BUN (36.9±11.0 mg/dL) and serum creatinine (0.8 mg/dL), which indicated the initiation of kidney injury. The loss of renal function peaked at 48 hours of reperfusion time (BUN 214.2±70.3 mg/dL and creatinine 1.83±1.22 mg/dL) and started to decline at 72 hours of reperfusion (data not shown). At 1 week of reperfusion, the renal function was significantly recovered, showing BUN of 104.2±45.6 mg/dL and serum creatinine of 1.11±0.36 mg/dL. This model provided a reversible or recoverable model of renal IRI that permitted the analysis of metabolic changes during initial injury, peak injury, and recovery phases.

### The global change of metabolites after renal ischemia/reperfusion

**Figure 2:**
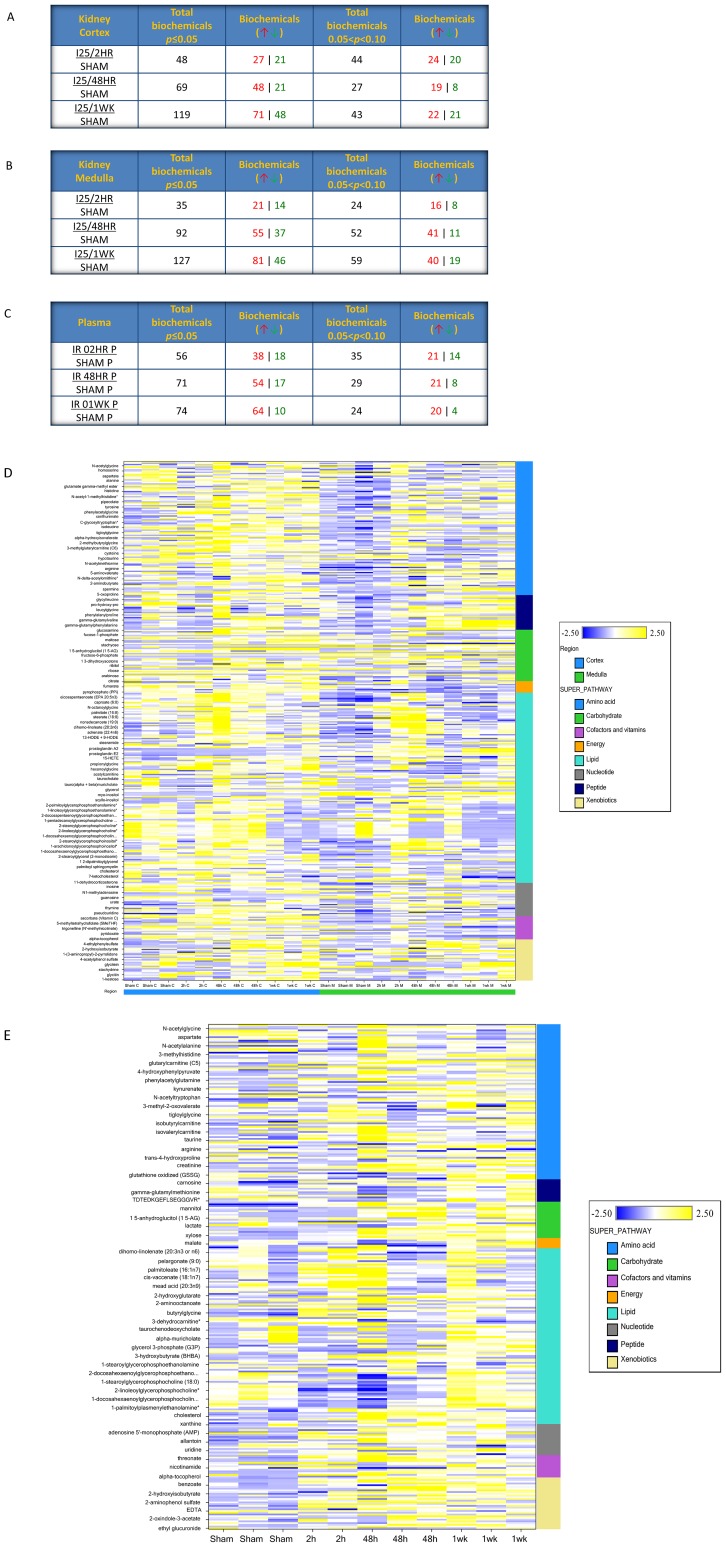
The global change of metabolites in ischemic acute kidney injury. Littermate C57BL/6 mice were subjected to sham operation or 25 minutes of bilateral renal ischemia with 2 hours, 48 hours, and 1 week of reperfusion. The renal cortex, renal medulla, and the plasma samples were collected at sacrifice for metabolites profiling. (A) The global change of metabolites in the kidney cortex; (B) The global change of metabolites in the kidney medulla; (C) The global change of metabolites in the plasma; (D) The heat map of the metabolites in the kidney cortex and medulla; (E) The heat map of the metabolites in the plasma.

Kidney cortex, medulla, and plasma samples were collected from the mice experienced sham operation or 25 minutes of bilateral renal ischemia with 2 hours, 48 hours and 1 week of reperfusion. The samples were processed for global metabolomics profiling using the gas chromatography/mass spectrometry (GC/MS) and liquid chromatography/mass spectrometry (LC/MS) platforms. The analysis detected 404 named chemicals from the kidney samples and 293 named chemical from the plasma samples. Following log transformation and imputation with minimum observed values for each compound, Welch's two-sample t-test was used to identify the chemicals that differed significantly between the renal IRI groups and the sham group. A summary of the numbers of chemicals that achieved statistical significance (p≤0.05), as well as those approaching significance (0.05<p<0.10), is shown in Fig. 2A, B and C for different matrices respectively.

The sample intensity in the kidney samples (Fig. 2D) and plasma samples (Fig. 2E) were indicated by the heat maps. Overall, the medulla metabolite levels were lower than those in the cortex in the sham condition. The ischemia/reperfusion effects was generally stronger in the cortex, with some effects shared and some distinct between these two kidney sub-regions. In the plasma samples, the global metabolites levels were elevated following renal ischemia/reperfusion with some metabolite classes showing early, and sometimes transient, changes.

### Early response of metabolites to renal ischemia/reperfusion

**Figure 3:**
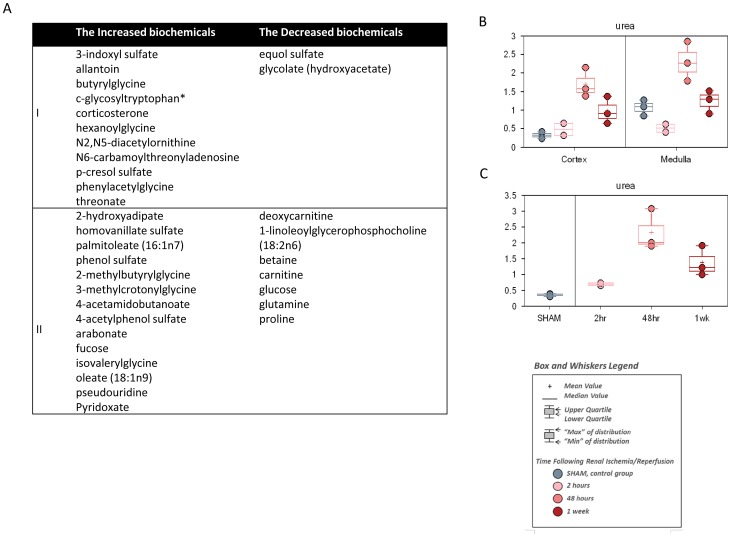
The metabolites of early response in renal IRI. C57BL/6 mice were subjected to sham operation or 25 minutes of bilateral renal ischemia with 2 hours, 48 hours, and 1 week of reperfusion. The renal cortex, renal medulla, and plasma samples were collected at sacrifice for metabolite profiling. (A) The increased or decreased chemicals in the kidney and the plasma at 2 hour reperfusion time. Group I: The chemicals showed significantly changes in both kidney and plasma (P<0.05). Group II: The chemicals were significantly changed in either kidney or plasma (P<0.05), but less significantly changed in the other (0.05<P<0.1); (B) Box plot showing the change of urea in kidney cortex and medulla; (C) Box plot showing the change of urea in plasma.

**Figure 4:**
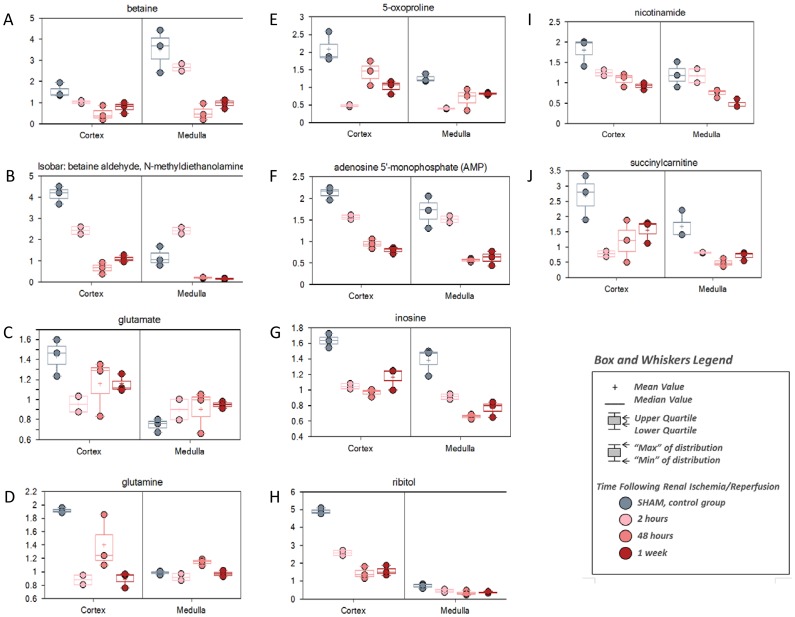
The metabolites of continuous inhibition in kidney during renal IRI. C57BL/6 mice were subjected to sham operation or 25 minutes of bilateral renal ischemia with 2 hours, 48 hours, and 1 week of reperfusion. The renal cortex and renal medulla were collected at sacrifice for metabolites profiling. The change of the metabolites was shown by box plots. (A) betaine; (B) Isobar: betaine aldehyde, N-methyldiethanolamine; (C) glutamate; (D) glutamine; (E) 5-oxoproline; (F) adenosine 5′-monophosphate (AMP); (G) inosine; (H) ribitol; (I) nicotinamide; (J) succiylcarnitine.

After comparing the samples of 2 hour reperfusion and sham, chemicals across classes were identified to show early response to ischemia/reperfusion injury (Fig. 3 & Fig. 4). Overall, there were 11 chemicals showing significant early elevations in both kidney and plasma (Fig. 3A). In addition, 14 chemicals were significantly increased in either kidney or plasma, with less significant increases in others (0.05<P<0.10) (Fig. 3A). Those chemicals included several specific amino acid and fatty acid catabolites, notably the acetylated, glycine-conjugated, and sulfated forms which were typically generated for urinary excretion. Among them, the chemicals such as phenylacetylglycine, isovalerylglycine, hexanoylglycine, were intermediates in the branched-chain amino acid catabolism. However, predominantly the glycine-conjugates but not the carnitine conjugates of the intermediates in the branched-chain amino acid catabolism pathway showed increase at 2 hour reperfusion time, such as 2-methylbutyrlglycine and 3-methylcrotonylglycine, as well as 3-indoxyl sulfate, phenol sulfate, hexanoylglycine and the vitamin (pyridoxate, the predominant excreted form of B6). Likewise, both plasma and kidney showed elevation of urea (Fig. 3B & C), with this pattern slightly delayed in the kidney medulla. In plasma and kidney, the early response also included the significant elevation that was sustained to some degree throughout the experimental time course, for the adrenal glucocorticoid, corticosterone (Fig. 3A), a primary component of the murine stress response and a strong lipid signature.

**Figure 5:**
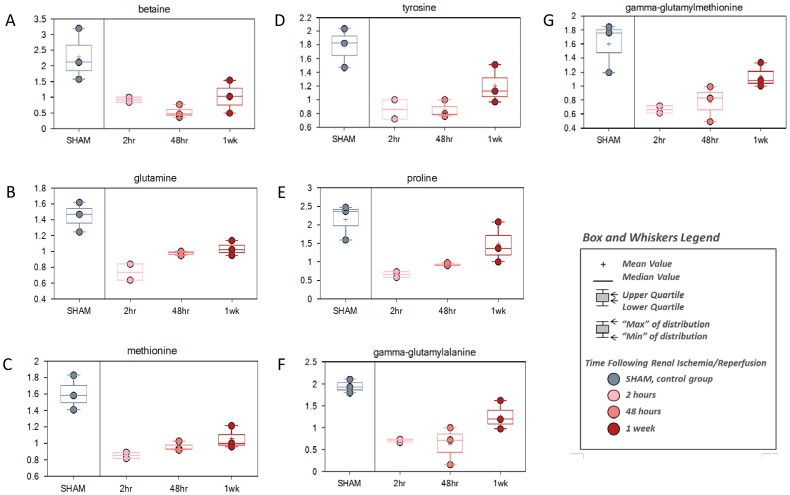
The metabolites of continuous inhibition in plasma after renal IRI. C57BL/6 mice were subjected to sham operation or 25 minutes of bilateral renal ischemia with 2 hours, 48 hours, and 1 week of reperfusion. The plasma samples were collected at sacrifice for metabolites profiling. The change of the metabolites was shown by box plots. (A) betaine; (B) glutamine; (C) methionine; (D) tyrosine; (E) proline; (F) gamma-glutamylalanine; (F) gamma-glutamylmethionine.

The chemicals showing decreased levels at 2 hour reperfusion time included a relatively high number of diet-derived or likely diet-derived chemicals, including xenobiotics equol sulfate and glycolate (Fig 3A). The depression in diet-derived metabolites at the early reperfusion time was probably due to the less food uptake during the recovery time from surgery and anesthesia, because the early depression was followed by the recovery to the normal levels or significantly above at later time points. But some metabolites showed a continuous or sustained decrease in kidney tissues in all reperfusion time points (Fig. 4), which was indicative of the impaired metabolism in the kidney of some specific amino acids, nucleotides, vitamin and energy, including betaine and Isobar: betaine aldehyde, N-methyldiethanolamine for glycine, serine and threonine metabolism; glutamate and glutamine for glutamate metabolism; 5-oxoproline for glutathione metabolism; adenosine 5′-monophosphate (AMP) for purine metabolism [adenine containing]; inosine for purine metabolism [(hypo)xanthine/inosine containing); ribitol for nucleotide sugars, pentose metabolism; nicotinamide for nicotinate and nicotinamide metabolism; and succinylcarnitine for Krebs cycle and energy metabolism. Of note, similar impaired amino acids metabolism also existed in the plasma, shown by the consistent decrease of several amino acids and peptides (Fig. 5), such as betaine, glutamine, methionine, tyrosine, proline, gamma-glutamylalanine, and gamma-glutamylmethionine.

In addition to their pathological roles in kidney injury, those significantly changed chemicals especially those changed in serum could be potential new diagnostic biomarkers since the current routinely used biomarkers (BUN and creatinine) do not show significantly induction at such early injury period (Fig. 1). For example, 3-indoxyl sulfate showed significantly increase in both kidney and serum at 2 hr reperfusion time and it was also reported to increase in other AKI models as well as in AKI patients [4], [11], [18]. In addition, 3-indoxyl sulfate has been used as an indicator to show the chronic kidney disease progression in hemodialysis patient [19]. Certainly further studies are necessary to confirm the early induction of 3-indoxyl sulfate in various AKI condition and the specificity of its induction in kidney injury. Finally, as a uremic toxin, 3-indoxyl sulfate showed toxicity to the human endothelial progenitor cells and renal proximal tubular cells, which suggests a potential remedy of AKI by the blockage of 3-indoxyl sulfate induction [18], [20]–[24].

### Perturbed energy metabolism in both plasma and kidney

Renal IRI leads to an abrupt decline of energy supply in kidney due to the lack of oxygen and nutrient supply, and mitochondrial damage [25]. Therefore, the response to ischemia/reperfusion encompassed several aspects of energy metabolism with signatures in both plasma and kidney tissue. Among this group of chemicals were clear distinctions between kidney cortex and kidney medulla. Glucose, free fatty acids, and amino acids serve as potential cellular energy sources under different conditions. Glucose metabolism is largely reflected by the activity of glycolysis/gluconeogenesis and the TCA cycle while the free fatty acids contribute to energy metabolism via fatty acid beta-oxidation and the TCA cycle. Under relatively extreme fasting conditions, amino acids from the protein breakdown also can contribute to the TCA cycle. In this study, complex changes over time and across matrices were observed for intermediates in these pathways.

**Figure 6:**
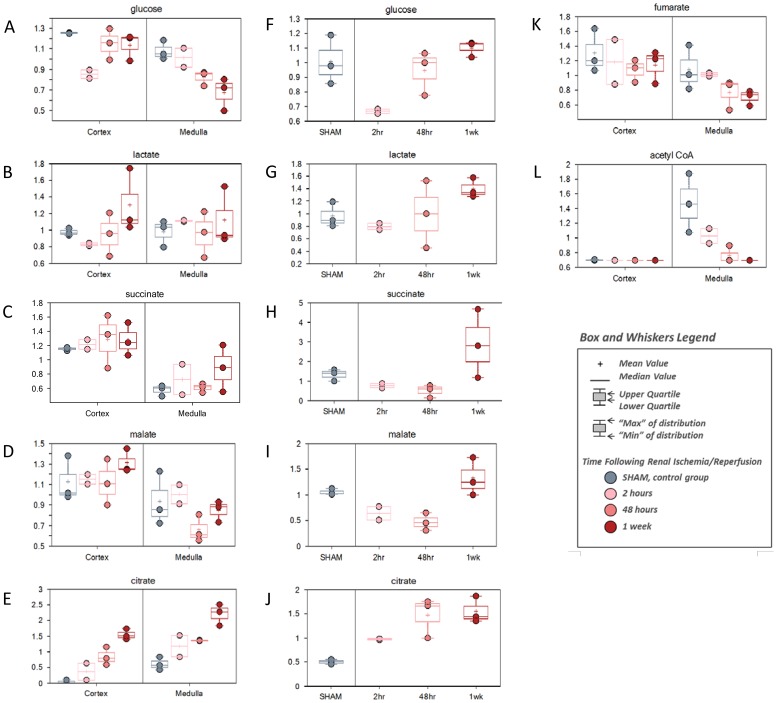
The change of metabolites involved in glucose metabolism and TCA cycle. C57BL/6 mice were subjected to sham operation or 25 minutes of bilateral renal ischemia with 2 hours, 48 hours, and 1 week of reperfusion. The renal cortex, renal medulla and plasma samples were collected at sacrifice for metabolites profiling. The change of the metabolites was shown by box plots. (A) glucose in kidney; (B) lactate in kidney; (C) succinate in kidney; (D) malate in kidney; (E) citrate in kidney; (F) glucose in plasma; (G) lactate in plasma; (H) succinate in plasma; (I) malate in plasma; (J) citrate in plasma; (K) fumarate in kidney; (L) acetyl CoA in kidney.

For glucose metabolism, the kidney cortex and the plasma samples showed early decreases in both glucose (Fig. 6A, F), and the metabolite of pyruvate (the glycolysis product), lactate (Fig. 6B, G), but recovery to near-sham levels by the 1 week reperfusion time. The intermediates in the TCA cycle, including succinate and malate showed a similar pattern in the plasma but not in the kidney cortex (Fig. 6 C, D, H, I). This pattern of change suggests a transient decrease in the use of glucose for energy metabolism in the kidney cortex and perhaps with systemic effects such that a similar change also registered in the plasma. By contrast, kidney medulla showed a slower decrease over time from sham to 1 week reperfusion in glycolysis (glucose and acetyl-CoA) and TCA cycle activity (fumarate) (Fig. 6K, L). Together, these changes supported a delayed energy metabolism response but longer-term negative energy status in the kidney medulla following renal IRI. In all three matrices, the TCA cycle intermediate and fatty acid biosynthetic precursor, citrate, showed a unique pattern of generally increasing levels from sham through the ischemia/reperfusion time course (Fig. 6E, J). Notably, previous reports have suggested that elevated citrate levels in urine correlate with reperfusion injury [26]. Accumulating citrate levels may be indicative of a block to TCA cycle progression and may contribute to the reduced glucose metabolism as well.

**Figure 7:**
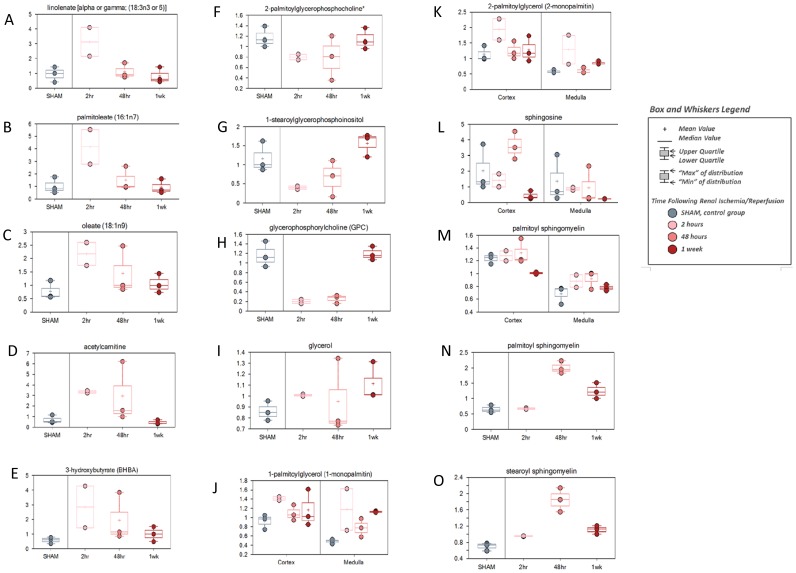
The change of metabolites involved in lipid metabolism. C57BL/6 mice were subjected to sham operation or 25 minutes of bilateral renal ischemia with 2 hours, 48 hours, and 1 week of reperfusion. The renal cortex, renal medulla and plasma samples were collected at sacrifice for metabolites profiling. The change of the metabolites was shown by box plots. (A) linolenate [alpha or gamma: (18∶3n3 or 6)] in plasma; (B) palmitoleate (16∶1n7) in plasma; (C) oleate (18∶1n9) in plasma; (D) acetylcamitine in plasma; (E) 3-hydroxybutyrate (BHBA) in plasma; (F) 2-palmitoylglycerophosphocholine* in plasma; (G) 1-stearoylglycerophosphoinositol in plasma; (H) glycerophosphorylcholine (GPC) in plasma; (I) glycerol in plasma; (J) 1-palmitoylglycerol (1-monopalmitin) in kidney; (K) 2-palmitoylglycerol (2-monopalmitin) in kidney; (L) sphingosine in kidney; (M) palmitoyl sphingomyelin in kidney; (N) palmitoyl sphingomyelin in plasma; (O) stearoyl sphingomyelin in plasma.

In this study, each matrix showed a relatively unique lipid metabolism signature associated with renal ischemia/reperfusion. In addition to the free fatty acids used in fatty acid beta-oxidation, lipid energy metabolism signatures may involve multiple additional small molecule metabolite classes. In plasma, the lipid metabolism signature, particularly in conjunction with the glucose signature above, suggests a substrate switch from carbohydrate to lipid metabolism for energy at the early post-ischemia/reperfusion time point with gradual normalization over the remainder of the reperfusion time. Early, albeit not often statistically significant, elevations were observed for multiple plasma free fatty acids including, for example, linolenate (0.05<p<0.1), palmitoleate (0.05<p<0.1), and oleate (p<0.05) (Fig. 7A, B, C). A concomitant early increase in plasma acetylcarnitine (which often reflects acetyl-CoA levels), and the ketone bodies acetoacetate and 3-hydroxybutryate (BHBA) (which serve as markers of fatty acid beta-oxidation rates), implicate the potential increased free fatty acid availability and breakdown (Fig. 7D, E). Free fatty acid levels were influenced by the dietary fat uptake, and the rates biosynthesis and breakdown of lipid stores and membrane phospholipids. Plasma levels of many lysolipids- glycerophospholipids with a single acyl chain whose levels reflected membrane remodeling and phospholipid breakdown activity, such as 2-palmitoylglycerophosphocholine, 1-stearoylglycerophosphoinositol and glycerophosphocholine (GPC), a breakdown product of choline-conjugated membrane phospholipids (but also an organic osmolyte, see below), showed decreased levels early and then normalization over the remainder of the reperfusion time (Fig. 7F, G, H). This pattern of phospholipid breakdown products, essentially a mirror image of the free fatty acid pattern, suggests that membrane breakdown and remodeling activity was transiently inhibited following renal ischemia/reperfusion. Elevated levels, albeit non-significant, at the early time point for glycerol, which could indicate lipolysis [the breakdown of triacylglycerol (TAG) lipid stores], suggests TAGs as a source of increased free fatty acids (Fig. 7I). The plasma lipid signature indicates the possibility that an immediate reduction in fatty acid incorporation into membranes and increased lipolysis combined to the increase of free fatty acid availability which may provide a source of increased fat metabolism for energy in the early ischemia/reperfusion response. Kidney cortex and kidney medulla shared an early and relatively sustained elevation in free fatty acids and the ketone body, BHBA, following renal ischemia. As with plasma, in conjunction with the evidence suggesting perturbed use of glucose for energy, this pattern and the transient elevation observed at the 2 hour time point for monoacylglycerols, such as 1-palmitoylglycerol and 2-palmitoylglycerol, implicate an energy substrate switch in kidney tissue from sugar to fat as a possible component of the early ischemia/reperfusion response (Fig. 7J, K).

Using more lipid as the energy source may also exist in AKI patient, which was reported to have disturbed serum lipid metabolism characterized by the increase of triglycerides (TG) and an extreme decrease in HDL-cholesterol (HDL-C) [27]. Thus, the appropriate nutrition supply may be critical during the treatment of AKI to provide enough free fatty acid for energy consumption, which probably can prevent the cells from breaking down the cellular membranes to obtain enough free fatty acid and can reduce cell injury.

Furthermore, another group of lipids, sphingolipids, have been shown to be the stress responsive factors in ischemic AKI [28]–[30]. Sphinganine-1-phosphate was shown to protect kidneys from injury but its precusors such as sphingosine, ceramide and sphingomyelins could be pro-apototic [28]–[32]. In our study a few metabolites involved in sphinolipids metabolism (but not sphingonine-1-phosphate) were detected. The change of sphingolipids was not obvious during early kidney injury; however, at 1 week reperfusion time there was a significant drop of sphingosine and palmitoyl sphingomyelin (Fig. 7L, M), which was consistent with the recovery condition of the tissue. Meanwhile, there was enormous accumulation of palmitoyl sphingomyelin and stearoyl sphingomyelin in the plasma (Fig. 7N, O). Although it is not clear whether the induction of plasma sphingomyelins were renal originated, those factors may mediate potential injuries in other organs.

### Impaired purine metabolism in kidney during ischemic injury

**Figure 8:**
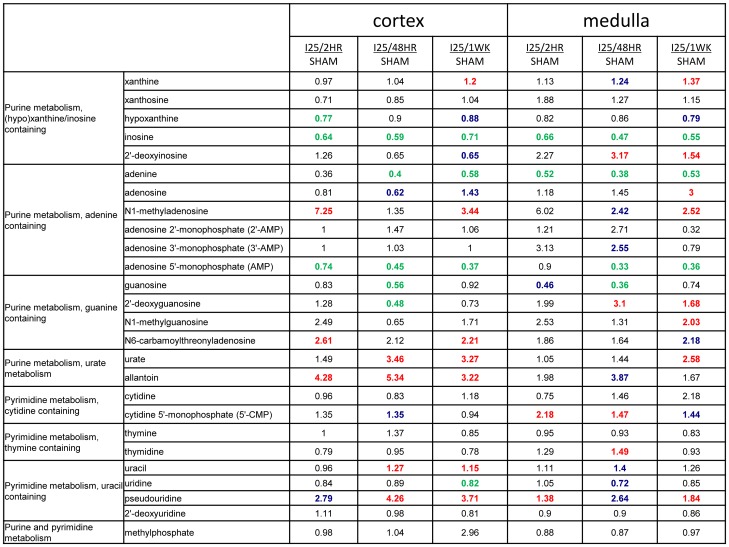
The change of metabolites in nucleotide metabolism. C57BL/6 mice were subjected to sham operation or 25 minutes of bilateral renal ischemia with 2 hours, 48 hours, and 1 week of reperfusion. The renal cortex and renal medulla were collected at sacrifice for metabolites profiling. The red colored numbers indicate statistically significant increase (P≤0.05). The green colored numbers indicate statistically significant decrease (P≤0.05). The blue colored numbers indicate narrowly missed statistical cutoff for significance (0.05<P<0.1).

The nucleotide metabolism has been noticed to be disturbed in ischemic injury for long time [33]. In our study, the impaired nucleotides metabolism, specifically the purine metabolism, was mainly shown in the kidney cortex and medulla, which was probably exhausted as alternative energy sources during injury, indicated by the early decrease of inosine and adenine, and the significant decrease of guanosine at 48 hour reperfusion time in the kidney (Fig. 8). The impaired purine metabolism in ischemic kidney injury was also reported before that the kidney injury could be ameliorated by the supplements of adenosine, inosine and guanosine [34]–[36]. However, no obvious impairment was detected for the pyrimidine metabolism such as cytidine, thymidine, and uridine (Fig. 8). In addition, the significant accumulation of urate in the kidney may result from the less excretion after kidney injury (Fig. 8), which could further induce hyperuricemia, though the increase of urate in plasma was detected at 48 hour and 1 week reperfusion time without statistically significance (data not shown).

### Changes in metabolites in osmotic regulation are evident in plasma and kidney by 48 hr reperfusion

**Figure 9:**
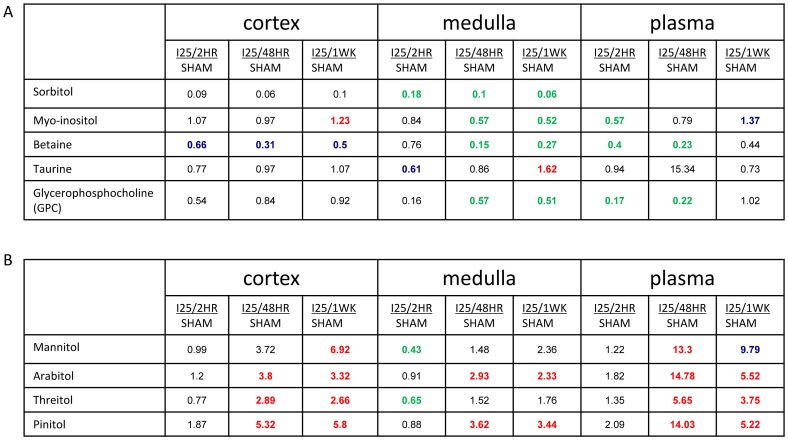
The change of metabolites involved in osmotic regulation. C57BL/6 mice were subjected to sham operation or 25 minutes of bilateral renal ischemia with 2 hours, 48 hours, and 1 week of reperfusion. The renal cortex, renal medulla and plasma samples were collected at sacrifice for metabolites profiling. The red colored numbers indicate statistically significant increase (P≤0.05). The green colored numbers indicate statistically significant decrease (P≤0.05). The blue colored numbers indicate narrowly missed statistical cutoff for significance (0.05<P<0.1). (A) The glucose derived polyols; (B) other polyols.

As a component of its normal function, particularly for the medulla, the kidney is exposed to osmotic extremes and is known to make use of small molecule organic osmolytes in maintaining osmotic balance. Among key players in osmotic regulation are the glucose-derived polyols sorbitol and inositol, betaine, taurine, and choline-derived glycerophosphocholine (GPC). The polyol pathway has been implicated as a component of renal injury induced by ischemic hind limb though the mechanism is unknown [37]. In our study, the small molecule osmolytes sorbitol, myoinositol, betaine, and GPC showed anticipated relative accumulation in kidney medulla compared to kidney cortex in the sham conditions and strong immediate and sustained decreased levels in kidney medulla following ischemia/reperfusion (Fig. 9A). In plasma, myoinositol and GPC also showed decreased levels at early time points but their levels returned to near-sham levels by one week. However, taurine did not show obvious decreases in all these three matrices. Overall, the time course of change in metabolite levels for multiple osmolytes indicates that the perturbation in osmotic regulation may be a key component to the ischemia/reperfusion metabolomic signature in both kidney medulla and plasma, which is sustained till one week reperfusion time in kidney. It is not surprising that the decline of renal function may affect the osmotic regulation. However, it would be interesting to speculate that the decrease in these osmolytes may also contribute to tissue damage.

However, there are multiple additional polyols with other patterns of change (Fig. 9B). For mannitol, arabitol, threitol, and pinitol, little difference was detected from sham at 2 hours post ischemia but significant elevation was evident in both plasma and kidney tissue by 48 hour reperfusion time. This kind of polyol elevation was sustained to the 1 week reperfusion time in kidney. The change of those polyols was unlike the osmolytes described above, but similar to the change patterns of diet-derived compounds. As the origin, function, and metabolic fates for many of those polyols remained poorly understood, it remains unclear if their change contributes to the osmolality regulation in renal IRI.

### Metabolic signature of inflammation in kidney tissue and plasma with ischemia/reperfusion

The metabolic signatures of inflammation associated with renal ischemia/reperfusion were evident in plasma and kidney tissue which is consistent with other studies showing the accumulation of immune cells in kidney after ischemic AKI [1], [38]. Metabolic pathways reporting on inflammation in this study included the generation of prostaglandins from omega-6 fatty acid precursors, the inflammatory cytokine-responsive kynurenine pathway for tryptophan degradation, and the generation of nitric oxide from arginine with citrulline as a byproduct.

**Figure 10:**
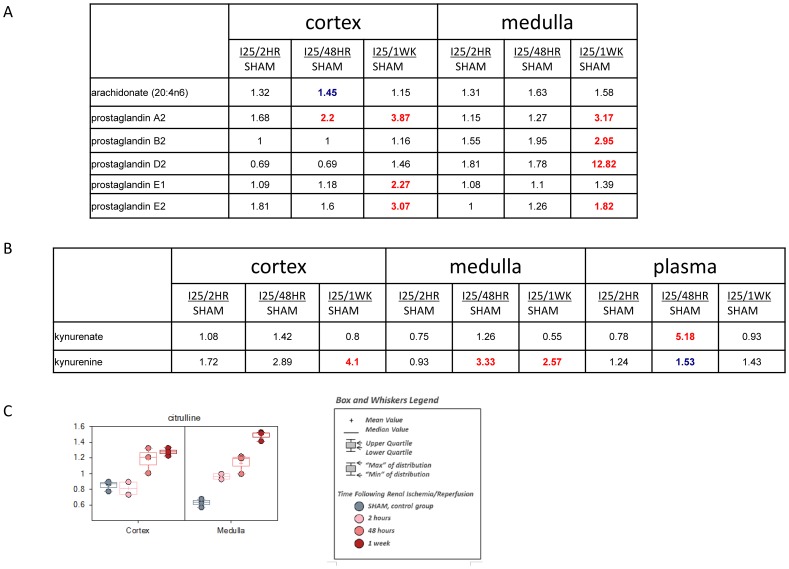
The change of metabolites involved in inflammation regulation. C57BL/6 mice were subjected to sham operation or 25 minutes of bilateral renal ischemia with 2 hours, 48 hours, and 1 week of reperfusion. The renal cortex, renal medulla and plasma samples were collected at sacrifice for metabolites profiling. The red colored numbers indicate statistically significant increase (P≤0.05). The green colored numbers indicate statistically significant decrease (P≤0.05). The blue colored numbers indicate narrowly missed statistical cutoff for significance (0.05<P<0.1). (A) The metabolites for the generation of prostaglandins in kidney; (B) The metabolites in tryptophan and kynurenine pathway; (C) The box plot showing the induction of citrulline in kidney.

However, the elevation of tissue levels for multiple prostaglandins was a relatively late event, appearing strongest at the 1 wk reperfusion time in kidney. Although the precursor arachidonate did not show significant induction, prostaglandin E1, prostaglandin B2, prostaglandin D2, prostaglandin E2, and prostaglandin A2 were elevated over time during reperfusion, a pattern consistent with the known inflammation component of the ischemia/reperfusion injury (Fig. 10A). The late induction of prostaglandins at one week reperfusion time suggests that they may exert more protective or wound healing role in ischemic AKI instead of promoting cell death and the cytoprotective effects of prostaglandins in ischemic AKI has been reported before [39]. Likewise, in both plasma and kidney tissue, the increased levels of the tryptophan metabolite kynurenine and its metabolite kynurenate with a more complex pattern further supported a pro-inflammatory environment, potentially systemically, following kidney ischemia and reperfusion (Fig. 10B). Arginine metabolism provides insight into nitrogen balance, proliferation, and inflammation. In this study, we identified significant accumulation of citrulline in kidney tissue, which could be produced by the inflammation-responsive generation of nitric oxide from arginine, indicating a strong possibility of inflammation (Fig. 10C & D). Although citrulline increase has been shown to indicate the development of end stage renal disease, its role in ischemic AKI is still not clear [40].

## Conclusions

In summary, renal ischemia/reperfusion produced strong, dynamic changes of metabolites in kidney cortex, kidney medulla, and plasma over time, which is highlighted by the early changes with strong elevation in both kidney tissues and plasma of many metabolites reporting on kidney function, such as uremic toxins, glycine-conjugated amino acids and fatty acids and compounds that share a dietary origin. During the most severely injured period (48 hour reperfusion time), the kidney and plasma showed evidence of altered energy metabolism affecting glycolysis, the TCA cycle and lipid metabolism with distinctions across matrices. The disturbed nucleotide metabolism was mainly indicated by the impaired purine metabolism but not the pyrimidine metabolism. Altered levels for several small molecule osmolytes support an influence of ischemia/reperfusion on osmoregulation that is apparent in kidney medulla and plasma, possibly due to the function loss of proximal tubules. The signatures of inflammation in kidney included significant elevation across multiple prostaglandins, increased catabolism of tryptophan along the kynurenate pathway, and the accumulation of citrulline in kidney. Tryptonphan and citrulline have been shown to be related to chronic kidney disease[40], [41], their roles in acute kidney injury require further research to clarify.

## References

[1] Bonventre JV, Yang L (2011) Cellular pathophysiology of ischemic acute kidney injury. J Clin Invest 121: 4210–4221. 22045571 10.1172/JCI45161 PMC3204829

[2] Weiss RH, Kim K (2012) Metabolomics in the study of kidney diseases. Nat Rev Nephrol 8: 22–33. 10.1038/nrneph.2011.152 22025087

[3] Zhang A, Sun H, Qiu S, Wang X (2013) Metabolomics insights into pathophysiological mechanisms of nephrology. Int Urol Nephrol. 10.1007/s11255-013-0600-2 24217804

[4] Uehara T, Horinouchi A, Morikawa Y, Tonomura Y, Minami K, et al.. (2013) Identification of metabolomic biomarkers for drug-induced acute kidney injury in rats. J Appl Toxicol. 10.1002/jat.2933 24114878

[5] Noto A, Cibecchini F, Fanos V, Mussap M (2013) NGAL and metabolomics: the single biomarker to reveal the metabolome alterations in kidney injury. Biomed Res Int 2013: 612032. 23607092 10.1155/2013/612032 PMC3625560

[6] Hanna MH, Segar JL, Teesch LM, Kasper DC, Schaefer FS, et al (2013) Urinary metabolomic markers of aminoglycoside nephrotoxicity in newborn rats. Pediatr Res 73: 585–591. 23411940 10.1038/pr.2013.34 PMC3640567

[7] Schnackenberg LK, Sun J, Pence LM, Bhattacharyya S, Gamboa da Costa G, et al (2012) Metabolomics evaluation of hydroxyproline as a potential marker of melamine and cyanuric acid nephrotoxicity in male and female Fischer F344 rats. Food Chem Toxicol 50: 3978–3983. 22902825 10.1016/j.fct.2012.08.010

[8] Sun J, Shannon M, Ando Y, Schnackenberg LK, Khan NA, et al (2012) Serum metabolomic profiles from patients with acute kidney injury: a pilot study. J Chromatogr B Analyt Technol Biomed Life Sci 893–894: 107–113. 10.1016/j.jchromb.2012.02.042 PMC3325145 22429878

[9] Wang J, Zhou Y, Xu M, Rong R, Guo Y, et al (2011) Urinary metabolomics in monitoring acute tubular injury of renal allografts: a preliminary report. Transplant Proc 43: 3738–3742. 22172837 10.1016/j.transproceed.2011.08.109

[10] Atzori L, Mussap M, Noto A, Barberini L, Puddu M, et al (2011) Clinical metabolomics and urinary NGAL for the early prediction of chronic kidney disease in healthy adults born ELBW. J Matern Fetal Neonatal Med 24 Suppl 240–43. 21781002 10.3109/14767058.2011.606678

[11] Zgoda-Pols JR, Chowdhury S, Wirth M, Milburn MV, Alexander DC, et al (2011) Metabolomics analysis reveals elevation of 3-indoxyl sulfate in plasma and brain during chemically-induced acute kidney injury in mice: investigation of nicotinic acid receptor agonists. Toxicol Appl Pharmacol 255: 48–56. 21640743 10.1016/j.taap.2011.05.015

[12] Xie G, Zheng X, Qi X, Cao Y, Chi Y, et al (2010) Metabonomic evaluation of melamine-induced acute renal toxicity in rats. J Proteome Res 9: 125–133. 19476335 10.1021/pr900333h

[13] Portilla D, Schnackenberg L, Beger RD (2007) Metabolomics as an extension of proteomic analysis: study of acute kidney injury. Semin Nephrol 27: 609–620. 18061843 10.1016/j.semnephrol.2007.09.006 PMC2684501

[14] Liu Y, Yan S, Ji C, Dai W, Hu W, et al (2012) Metabolomic changes and protective effect of (L)-carnitine in rat kidney ischemia/reperfusion injury. Kidney Blood Press Res 35: 373–381. 22487906 10.1159/000336171

[15] Wei Q, Dong Z (2012) Mouse model of ischemic acute kidney injury: technical notes and tricks. Am J Physiol Renal Physiol 303: F1487–1494. 22993069 10.1152/ajprenal.00352.2012 PMC3532486

[16] Wei Q, Dong G, Chen JK, Ramesh G, Dong Z (2013) Bax and Bak have critical roles in ischemic acute kidney injury in global and proximal tubule-specific knockout mouse models. Kidney Int 84: 138–148. 23466994 10.1038/ki.2013.68 PMC3686831

[17] Zhang D, Liu Y, Wei Q, Huo Y, Liu K, et al.. (2014) Tubular p53 Regulates Multiple Genes to Mediate AKI. J Am Soc Nephrol. 10.1681/ASN.2013080902 PMC4178437 24700871

[18] Wu VC, Young GH, Huang PH, Lo SC, Wang KC, et al (2013) In acute kidney injury, indoxyl sulfate impairs human endothelial progenitor cells: modulation by statin. Angiogenesis 16: 609–624. 23408148 10.1007/s10456-013-9339-8

[19] Agatsuma S, Sekino H, Watanabe H (1996) Indoxyl-beta-D-glucuronide and 3-indoxyl sulfate in plasma of hemodialysis patients. Clin Nephrol 45: 250–256. 8861801

[20] Saito S, Shimizu H, Yisireyili M, Nishijima F, Enomoto A, et al (2014) Indoxyl Sulfate-Induced Activation of (Pro)Renin Receptor Is Involved in Expression of TGF-beta1 and alpha-Smooth Muscle Actin in Proximal Tubular Cells. Endocrinology 155: 1899–1907. 24601883 10.1210/en.2013-1937

[21] Yisireyili M, Shimizu H, Saito S, Enomoto A, Nishijima F, et al (2013) Indoxyl sulfate promotes cardiac fibrosis with enhanced oxidative stress in hypertensive rats. Life Sci 92: 1180–1185. 23702423 10.1016/j.lfs.2013.05.008

[22] Bolati D, Shimizu H, Yisireyili M, Nishijima F, Niwa T (2013) Indoxyl sulfate, a uremic toxin, downregulates renal expression of Nrf2 through activation of NF-kappaB. BMC Nephrol 14: 56. 23496811 10.1186/1471-2369-14-56 PMC3599003

[23] Shimizu H, Yisireyili M, Nishijima F, Niwa T (2013) Indoxyl sulfate enhances p53-TGF-beta1-Smad3 pathway in proximal tubular cells. Am J Nephrol 37: 97–103. 23363842 10.1159/000346420

[24] Shimizu H, Yisireyili M, Higashiyama Y, Nishijima F, Niwa T (2013) Indoxyl sulfate upregulates renal expression of ICAM-1 via production of ROS and activation of NF-kappaB and p53 in proximal tubular cells. Life Sci 92: 143–148. 23201429 10.1016/j.lfs.2012.11.012

[25] Zhan M, Brooks C, Liu F, Sun L, Dong Z (2013) Mitochondrial dynamics: regulatory mechanisms and emerging role in renal pathophysiology. Kidney Int 83: 568–581. 23325082 10.1038/ki.2012.441 PMC3612360

[26] Hauet T, Baumert H, Gibelin H, Godart C, Carretier M, et al (2000) Citrate, acetate and renal medullary osmolyte excretion in urine as predictor of renal changes after cold ischaemia and transplantation. Clin Chem Lab Med 38: 1093–1098. 11156334 10.1515/CCLM.2000.162

[27] Ogata F, Hirasawa Y, Takahashi S, Tajiri M, Kanno K, et al (1981) Disturbance of serum lipid metabolism in acute renal failure. Clin Exp Dial Apheresis 5: 361–371. 7341022 10.3109/08860228109076027

[28] Iwata M, Herrington J, Zager RA (1995) Sphingosine: a mediator of acute renal tubular injury and subsequent cytoresistance. Proc Natl Acad Sci U S A 92: 8970–8974. 7568054 10.1073/pnas.92.19.8970 PMC41089

[29] Zager RA, Conrad DS, Burkhart K (1998) Ceramide accumulation during oxidant renal tubular injury: mechanisms and potential consequences. J Am Soc Nephrol 9: 1670–1680. 9727376 10.1681/ASN.V991670

[30] Zager RA, Iwata M, Conrad DS, Burkhart KM, Igarashi Y (1997) Altered ceramide and sphingosine expression during the induction phase of ischemic acute renal failure. Kidney Int 52: 60–70. 9211347 10.1038/ki.1997.304

[31] Woodcock J (2006) Sphingosine and ceramide signalling in apoptosis. IUBMB Life 58: 462–466. 16916783 10.1080/15216540600871118

[32] Park SW, Kim M, Chen SW, D'Agati VD, Lee HT (2010) Sphinganine-1-phosphate attenuates both hepatic and renal injury induced by hepatic ischemia and reperfusion in mice. Shock 33: 31–42. 19752779 10.1097/SHK.0b013e3181c02c1f PMC2794916

[33] Vary TC, Angelakos ET, Schaffer SW (1979) Relationship between adenine nucleotide metabolism and irreversible ischemic tissue damage in isolated perfused rat heart. Circ Res 45: 218–225. 445706 10.1161/01.res.45.2.218

[34] Lee HT, Emala CW (2001) Systemic adenosine given after ischemia protects renal function via A(2a) adenosine receptor activation. Am J Kidney Dis 38: 610–618. 11532695 10.1053/ajkd.2001.26888

[35] Fitzpatrick JM, Wallace DM, Whitfield HN, Watkinson LE, Fernando AR, et al (1981) Inosine in ischaemic renal surgery: long-term follow-up. Br J Urol 53: 524–527. 7317735 10.1111/j.1464-410x.1981.tb03253.x

[36] Kelly KJ, Plotkin Z, Vulgamott SL, Dagher PC (2003) P53 mediates the apoptotic response to GTP depletion after renal ischemia-reperfusion: protective role of a p53 inhibitor. J Am Soc Nephrol 14: 128–138. 12506145 10.1097/01.asn.0000040596.23073.01

[37] Yagihashi S, Mizukami H, Ogasawara S, Yamagishi S, Nukada H, et al (2010) The role of the polyol pathway in acute kidney injury caused by hindlimb ischaemia in mice. J Pathol 220: 530–541. 20112370 10.1002/path.2671

[38] Kinsey GR, Sharma R, Okusa MD (2013) Regulatory T cells in AKI. J Am Soc Nephrol 24: 1720–1726. 24136922 10.1681/ASN.2013050502 PMC3810092

[39] Paller MS, Manivel JC (1992) Prostaglandins protect kidneys against ischemic and toxic injury by a cellular effect. Kidney Int 42: 1345–1354. 1474766 10.1038/ki.1992.426

[40] Lau T, Owen W, Yu YM, Noviski N, Lyons J, et al (2000) Arginine, citrulline, and nitric oxide metabolism in end-stage renal disease patients. J Clin Invest 105: 1217–1225. 10791996 10.1172/JCI7199 PMC315437

[41] Schulman G (2012) A nexus of progression of chronic kidney disease: tryptophan, profibrotic cytokines, and charcoal. J Ren Nutr 22: 107–113. 22200426 10.1053/j.jrn.2011.10.035

